# Proteomic Determinants of Muscle Size in Older Men: The Osteoporotic Fractures in Men Study

**DOI:** 10.1093/geroni/igaf122.471

**Published:** 2025-12-31

**Authors:** Peggy Cawthon, Tyler Mansfield, Jodi Lapidus, William Evans, Daniel Evans, Eric Orwoll

**Affiliations:** California Pacific Medical Center, San Francisco, California, United States; California Pacific Medical Center, San Francisco, California, United States; Oregon Health & Science University, Portland, Oregon, United States; University of California, Berkeley, Berkeley, California, United States; University of California, San Francisco, San Francisco, California, United States; Oregon Health & Science University, Portland, Oregon, United States

## Abstract

In MrOS, men with low muscle mass as determined by deuterated creatine dilution (D3Cr muscle mass) are weaker, slower, and have a higher risk of incident falls, mobility limitation, disability, fractures and death. Proteomic determinants of D3Cr muscle mass are not known. Serum proteomics were assayed using the SOMAlogic 7K panel. Of the 7166 aptamers included in analyses, 475 were significantly associated with d3Cr muscle after adjusting for age and weight (adjusted p < 0.05). In age and weight adjusted models controlling for multiple comparisons, we found significant positive associations between D3Cr muscle mass with creatine kinase M type, and creatine kinase M type:B type heterodimer (r > 0.34, adjusted p < 3x10^(-10) for both). These positive correlations are expected, as the D3Cr dilution approach relies on assessment of total body creatine pool size, thus, relative abundance of creatine kinase should be related to D3Cr muscle mass given the properties of this assay. When controlling for multiple comparisons, we also found significant negative correlations between D3Cr muscle mass and SVEP1 (protein with a role in vascular disease) and brorin (a bone morphogenic protein) (r<-0.23, adjusted p < 3x10^(-7) for all); these may represent new targets to identify or target low muscle mass. Leptin and FSTL3 were negatively correlated with D3Cr muscle mass (r<-0.25, adjusted p < 6x10^(-6) for both). Further work will identify pathways rather than specific proteins that relate to low D3Cr muscle mass, and whether these associations replicate in other populations and in women.

